# Cadmium accumulation is enhanced by ammonium compared to nitrate in two hyperaccumulators, without affecting speciation

**DOI:** 10.1093/jxb/erw270

**Published:** 2016-07-06

**Authors:** Miaomiao Cheng, Peng Wang, Peter M. Kopittke, Anan Wang, Peter W.G. Sale, Caixian Tang

**Affiliations:** ^1^Department of Animal, Plant and Soil Sciences, Centre for AgriBioscience, La Trobe University, Victoria 3086, Australia; ^2^School of Agriculture and Food Sciences, The University of Queensland, Queensland 4072, Australia

**Keywords:** *Carpobrotus rossii*, Cd speciation, Cd translocation, halophytes, nitrogen form, phytoremediation, *Solanum nigrum*, synchrotron, XANES.

## Abstract

Nitrogen fertilization could improve the efficiency of Cd phytoextraction in contaminated soil and thus shorten the remediation time. However, limited information is available on the effect of N form on Cd phytoextraction and associated mechanisms in plants. This study examined the effect of N form on Cd accumulation, translocation, and speciation in *Carpobrotus rossii* and *Solanum nigrum*. Plants were grown in nutrient solution with 5–15 μM Cd in the presence of 1000 µM NH_4_^+^ or NO_3_^−^. Plant growth and Cd uptake were measured, and Cd speciation was analyzed using synchrotron-based X-ray absorption spectroscopy. Shoot Cd accumulation was 30% greater with NH_4_^+^ than NO_3_^−^ supply. *Carpobrotus rossii* accumulated three times more Cd than *S. nigrum.* However, Cd speciation in the plants was not influenced by N form, but it did vary with species and tissues. In *C. rossii*, up to 91% of Cd was bound to S-containing ligands in all tissues except the xylem sap where 87–95% were Cd-OH complexes. Furthermore, the proportion of Cd-S in shoots was substantially lower in *S. nigrum* (44–69%) than in *C. rossii* (60–91%). It is concluded that the application of NH_4_^+^ (instead of NO_3_^−^) increased shoot Cd accumulation by increasing uptake and translocation, rather than changing Cd speciation, and is potentially an effective approach for increasing Cd phytoextraction.

## Highlight

Supply of NH_4_^+^ compared with NO_3_^−^ increased plant Cd uptake, translocation and accumulation in *Carpobrotus rossii* and *Solanum nigrum*. Cd-S was the dominant Cd species, with Cd being transported as free ions in xylem.

## Introduction

Although widespread in the environment, cadmium (Cd) is a non-essential element with no known physiological functions. It enters soil from a variety of sources including application of metal-containing sewage sludge, phosphate fertilizers, waste from incinerators, and other industrial wastes (Nicholson *et al.*, 1994). Cadmium presents a risk because it accumulates readily in plants to levels that are harmful in animal and human diets. It has been recognized that due to the efficient soil-to-plant transfer of Cd, dietary intake of food from crops grown on Cd-contaminated soils is a major route of Cd exposure to human health (WHO, 2010). Therefore, effective ways to remediate Cd-contaminated soils and mitigate Cd accumulation in crops are needed.

Among the various approaches for remediation, phytoextraction has attracted substantial attention because it is cost-efficient and environmentally friendly (Mahar *et al.*, 2016). Phytoextraction reduces metal concentrations in contaminated soils through the accumulation of these metals in the above-ground biomass of plants. For this purpose, hyperaccumulators are plants that can accumulate metals to levels at least 100 times that of most plant species (Baker and Brooks, 1989). Although many studies have emphasized the use of plants for remediation of Cd-contaminated soils, the main limitation of the phytoextraction approach is the long remediation time. To overcome this limitation, various strategies have been used to increase shoot biomass or increase the metal concentration in the harvestable portions of the plants.

Nitrogen (N) fertilization is an agronomic strategy to improve phytoextraction. As an essential nutrient, N fertilization increases shoot biomass of N-deficient plants, with this also potentially increasing Cd accumulation. However, the effect of N on Cd accumulation varies with the N source and the application rate (Mitchell *et al.*, 2000; Maier *et al.*, 2002; Wångstrand *et al.*, 2007). Nitrate (NO_3_^−^) and ammonium (NH_4_^+^) are the main sources of inorganic N taken up by plants, with N uptake comprising up to 80% of the total ion uptake. Therefore, the form of N supply plays an important role in the cation–anion balance, cellular pH, and rhizospheric pH (Marschner, 2011). For NH_4_^+^, uptake results in an excess of cations over anions, with net extrusion of protons and acidification of the rhizosphere –potentially increasing the availability of toxic metals such as Cd (Wu *et al.*, 1989). The opposite is true for NO_3_^−^, with uptake resulting in an excess of anions over cations. However, some studies have actually shown that supply of NO_3_^−^ (rather than NH_4_^+^) increases uptake of Cd and other metals. For example, supply of NO_3_^−^ enhanced Cd and Zn accumulation in *Noccaea caerulescens* (formerly *Thlaspi caerulescens*) compared to NH_4_^+^, even though NH_4_^+^ lowered rhizoshphere pH (Monsant *et al.*, 2008; Xie *et al.*, 2009). Similarly, using nutrient solutions, supply of NO_3_^−^ increased Cd uptake in tomato (*Solanum lycopersicum*), potato (*S. tuberosum*), and in the hyperaccumulators *N. caerulescens* and *Sedum plumbizincicola* (Xie *et al.*, 2009; Luo *et al.*, 2012; Hu *et al.*, 2013; Jönsson and Asp, 2013). These findings indicate that, other than just influencing rhizosphere pH, N form also influences Cd uptake through additional mechanisms.

It is possible that the N form also influences Cd uptake and accumulation through changes in root morphology and plant growth (Rosen *et al.*, 1990; Bloom *et al.*, 2002) or through changes in the membrane potential of root cells (the driving force for cation uptake). For example, it has been reported that the uptake of NH_4_^+^ causes depolarization of cell membranes and thus reduces Cd uptake, whilst NO_3_^−^ favors Cd uptake by hyperpolarizing membrane potential (McClure *et al.*, 1990; Miller *et al.*, 2001; Zaccheo *et al.*, 2006). Furthermore, the N form may influence the expression of cation transporters, which may in turn alter Cd uptake and translocation given that Cd uptake is probably via the transport systems of other cations (Clemens *et al.*, 2002; Luo *et al.*, 2012).

The form of N supply may also alter the chemical speciation of Cd in plants, thereby influencing Cd translocation and accumulation. It is known that most transition metal ions in plants, including Cd, are bound by various ligands rather than being present as hydrated ions, and the chelation of Cd in plants determines its sequestration and mobility (Clemens, 2006). Moreover, uptake and assimilation of different N forms influence the production of amino acids and organic acids in plants, which in turn impacts the ligands for Cd complexation. Compared to NH_4_^+^, NO_3_^−^ increases the concentrations of organic acids but decreases amino acids (Roosta and Schjoerring, 2007; Marschner, 2011; White-Monsant and Tang, 2013). Furthermore, the form of N supplied influences the distribution of organic compounds in plant tissues, with NH_4_^+^ mainly being assimilated in roots whilst NO_3_^−^ assimilates in both roots and shoots (Marschner, 2011). It has been reported that amino acids, peptides, proteins, and organic acids are main ligands for Cd complexation, and that Cd speciation in plants varies with species, tissues, and environmental conditions (Salt *et al.*, 1995, 1997; Vogel-Mikuš *et al.*, 2010; Tian *et al.*, 2011). However, it remains unclear how the N form influences the speciation of Cd within plant tissues – which in turn will influence Cd uptake, translocation and hence accumulation.

The present study aimed to examine the effect of N form on Cd uptake and accumulation in two Australian native plant species, *Carpobrotus rossii* and *Solanum nigrum*, with both species having shown potential for Cd phytoextraction (Wei *et al.*, 2013; Zhang *et al.*, 2014). Specifically, this study aimed to determine whether the form of N supply alters Cd accumulation and speciation in root and shoot tissues. It was hypothesized that the supply of nitrate, relative to ammonium, would increase Cd uptake and accumulation, and that the increased Cd accumulation would be associated with changes in Cd speciation within differing tissues. Synchrotron-based X-ray absorption spectroscopy was used for *in situ* analyses of Cd speciation in the tissues.

## Materials and methods

### Plant growth

*Carpobrotus rossii* (Haw.) Schwantes (Aizoaceae) was collected from a rural landfill site (37°36′S, 143°35′E, Snake Valley, Shire of Pyrenees) in Victoria, Australia, while seeds of *Solanum nigrum* L. were collected from plants grown on the La Trobe University farm (37°72′S, 145°4′E). Seeds of *S. nigrum* were germinated in a solution containing 600 µM CaCl_2_ and 5 µM H_3_BO_3_ in a dark controlled-environment room for 5 d. For *C. rossii*, uniform cuttings (two nodes each cutting) were washed with tap water. The plant materials were transplanted to 5-l polyethylene pots filled with a basal nutrient solution aerated continuously. The basal nutrient solution had the following composition (µM): 500 NH_4_NO_3_; 200 MgSO_4_; 10 KH_2_PO_4_; 600 K_2_SO_4_; 600 CaCl_2_; 20 FeNaEDTA; 5 H_3_BO_3_; 1 MnSO_4_; 0.2 CuSO_4_; 0.03 Na_2_MoO_4_; 1 ZnSO_4_. The root systems were well-developed after grown in these solutions for 15 d (*C. rossii*) and 30 d (*S. nigrum*). After this growth in basal solutions, three seedlings of *C. rossii* and five of *S. nigrum* were transferred to new pots containing the treatment solutions (below).

The experiment consisted of eight treatments and was replicated three times. The eight treatments were two plant species (*C. rossii* and *S. nigrum*) × two forms of N (500 µM (NH_4_)_2_SO_4_ and 1000 µM KNO_3_) × two Cd concentrations (5 and 15 µM Cd). Solution pH was buffered with 2mM MES [2-(N-morpholino)ethane-sulphonic acid] and 1.2ml of 1M KOH was used to adjust pH to ca. 6.0, which was maintained daily using 1M KOH. Since sulfur (S) plays an important role in Cd binding, 500 µM K_2_SO_4_ was added to the KNO_3_ treatments in order to ensure constant S concentrations in all treatments [hence, the K concentration was 1210 µM in the (NH_4_)_2_SO_4_ treatments and 3210 µM in the KNO_3_ treatments]; the composition of other nutrients was the same as that of the basal nutrient solution. Solutions were renewed every 3 d. Plants were grown in a controlled-environment growth room with 14-h photoperiod, a light intensity of 400 μmol m^−2^ s^−1^, 20 °C during the day and 18 °C during the night, and 50% relative humidity during the day, and 60% relative humidity during the night.

### Xylem sap collection

After growth in the treatment solutions for 14 d, xylem sap was collected according to the method of Monsant *et al.* (2011). Briefly, the stem was cut with a fresh razor blade, with the root stump immediately washed with deionized water and dried with paper tissue. Xylem sap was collected for 1h using a micropipette and transferred into 1.5-ml Eppendorf tubes. The xylem sap samples from the various plants in each pot were pooled, with each treatment having three replicates. For *S. nigrum*, however, the volume of xylem sap collected was insufficient to permit analysis, and hence only samples from *C. rossii* seedlings are presented here.

### Plant analysis

After collecting the xylem sap, the plants were harvested and fresh weights recorded. Roots were divided into two parts, with the first subsample immersed in ice-cold 20mM Ca(NO_3_)_2_ for 15min, washed with deionized water, frozen in liquid nitrogen, and stored at −80 °C. The second root subsample was immersed in ice-cold 20mM Na_2_-EDTA for 15min to remove Cd adhering to the root surface and then washed with deionized water and weighed. The roots were examined using a root scanner at 600 dpi (Epson Perfection 4990 Scanner, model J131B, Epson Inc.) to determine morphological parameters (length and surface area) before being oven-dried (at 80 °C in paper bags) for analysis. After washing with deionized water, shoots were blot-dried and separated into stems, old leaves and young leaves. Like the roots, the shoots were subdivided into two subsamples. The first subsample was frozen in liquid nitrogen, and stored at −80 °C, with the second subsample oven-dried. For the plants grown in solutions containing 5 μM Cd, a portion of each tissue was also freeze-dried for X-ray absorption spectroscopy analysis. The oven-dried samples were ground and digested using HNO_3_ in a microwave digester (Multiwave 3000, Anton Paar). The concentrations of elements in the digests and xylem sap (0.5ml mixed with 2.5ml of 5% HNO_3_) were analyzed using an inductively coupled plasma optical emission spectrometry (ICP-OES) (Perkin Elmer Optima 8000, MA, USA).

### Cadmium speciation by X-ray absorption spectroscopy (XAS)

The speciation of Cd in different tissues was examined at the XAS beamline of the Australian Synchrotron (Victoria, Australia). The energy of each spectrum was calibrated by simultaneous measurement, in transmission, of a metallic Cd foil reference (K-edge at 27 711eV). The spectra were collected in the fluorescence mode with a 100-element solid-state Ge detector. To minimize beam-induced artifacts and thermal disorder, samples were placed in a cryostat sample holder (maintained at ca. 12K, liquid helium). The beam size was adjusted to ca. 2×0.5mm.

To prepare the tissues for analysis, approximately 1–2g frozen or freeze-dried samples were ground under liquid nitrogen using an agate mortar and pestle. The homogenized tissues were placed into a sample holder with Kapton tape windows cooled with liquid nitrogen before being transferred directly to the cryostat for analysis. For the xylem sap, treated plants were transported to the Australian Synchrotron (ca. 45min) before growth for an additional 24h. The sap was thus collected immediately prior to analysis. The xylem sap was mixed in 30% glycerol (see below) and loaded into a sample holder sealed with Kapton tape.

A total of 14 Cd-containing standard compounds (eleven aqueous and three solids) were analyzed using Cd K-edge XANES (X-ray absorption near edge structure) spectroscopy to allow interpretation of the XANES spectra from the experimental tissue samples. The eleven aqueous standards consisted of (i) 1mM Cd(NO_3_)_2_, (ii–x) 1mM Cd(NO_3_)_2_ mixed with various ligands at a final concentration of 5mM [phytate, histidine, citrate, malate, succinate, cysteine, glutathione (GSH), methionine (MT), and phytochelatin 2 (PC2)], and (xi) 1mM Cd(NO_3_)_2_ mixed with 0.1% polygalacturonic acid – this being the major charged component of the cell wall. Standards were prepared using a 10mM Cd(NO_3_)_2_ stock solution together with 50mM solutions of the various ligands, or 1% polygalacturonic acid stock solution. All aqueous standards (other than polygalacturonic acid) were mixed in 30% glycerol to limit ice-crystal formation during cooling. The 1% of polygalacturonic acid stock solution, with 30.4 μmol ml^−1^ free carboxyl groups determined by titration with 0.02M NaOH to neutrality, was prepared approximately 24h before use and kept at 4 °C (Kopittke *et al.*, 2011; Taylor and Walter, 2012). The citrate, GSH, malate, phytate, succinate, histidine, and MT standards were adjusted to ca. pH 6, cysteine to pH 7, and PC2 to pH 10 using 0.1M NaOH, while pH was not adjusted for the polygalacturonic acid or the 1mM aqueous Cd(NO_3_)_2_. Where constants were available, GEOCHEM-EZ was used to model the standard solutions, with the results indicating that >97% of Cd was complexed with citric acid, 85% with succinic acid, malic acid, and histidine. The three solid standards, CdS, CdO, and CdCO_3_, were diluted to 100mg Cd kg^−1^ using cellulose.

Multiple XANES scans were performed for each sample, with two scans per standard, and either two or three scans for each plant tissue sample. Beam-induced damage was assessed using a tissue sample, with two XANES scans conducted at the same sample location – the two scans were then compared to see if they differed. For other samples, positions were changed after each scan to reduce the risk of beam damage and to obtain representative spectra.

The XANES spectrum for each sample was energy-normalized using the reference energy of the Cd foil, with replicate spectra for each sample merged using Athena (version 0.9.22) (Ravel and Newville, 2005). Principal component analysis (PCA) of the normalized sample spectra was employed to assess how many independent components were contained in the samples, and target transformation (TT) was used to identify relevant standards for linear combination fitting (LCF) of the sample spectra. PCA and TT were undertaken using SixPack (Webb, 2005). Using PCA, the XANES spectra of the plant tissues were compared with those of the standards to evaluate the number of relevant components indicated by the indicator function (IND) reaching a minimum. The results of PCA indicated that the first four components accounted for 98.6% of the total variance of the XANES spectra, so only four components were needed to fit the data. Target transformation was then used to identify the standard spectra in the LCF analyses by selecting standards with a SPOIL value <3. Nine of the 14 reference spectra met this criterion, these being Cd-citrate, Cd-malate, Cd-succinate, Cd-polygalaturonate, Cd-GSH, Cd-cysteine, Cd-PC2, Cd(NO_3_)_2_, and CdS (Supplementary Table S1 at *JXB* online). The LCF was used to identify the relative proportions of standard spectra within the sample spectra as the XANES spectrum represents a combination of all Cd species present in the portion of sample transected by the beam, and the fitting energy range was –30 to +100eV relative to the Cd K-edge.

### Statistical analysis

The effect of N form and Cd concentration in solution on plant biomass, tissue Cd concentrations, shoot Cd content, and Cd translocation was examined using a two-way analysis of variance for each species. Significant (*P*≤0.05) differences between means were identified by Tukey’s HSD test using GenStat v. 11 (VSN international).

## Results

### Plant growth

**Fig. 1.**
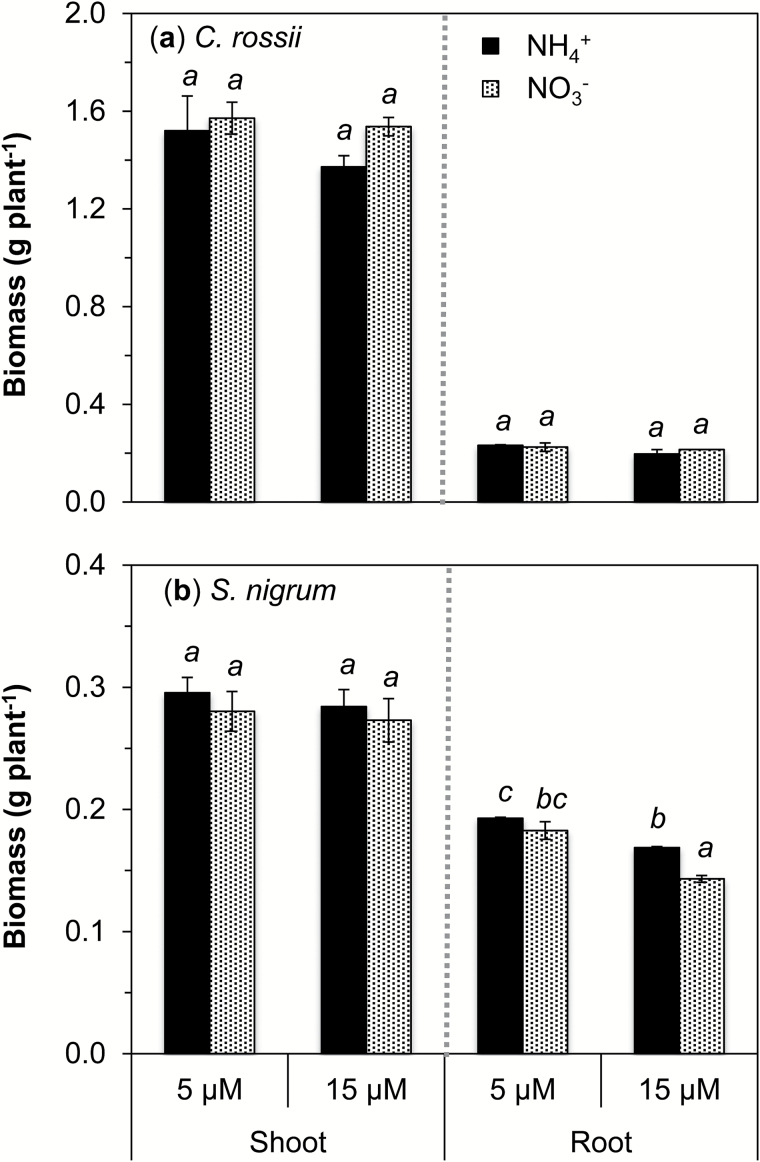
Effects of N form supplied on dry weights of *Carpobrotus rossii* (a) and *Solanum nigrum* (b) grown for 14 d in solutions containing Cd at a concentration of either 5 or 15 µM. Bars represent the standard errors (*n*=3). For each panel, different letters above the bars indicate significant differences among treatments for each tissue of individual species (Tukey’s test, *P*<0.05).

All plants appeared healthy during the experimental period, with little visual difference between treatments. Overall, *C. rossii* had shoot biomass 5-fold higher than *S. nigrum* (Fig. 1). Despite this overall difference in biomass production between the two species, the form of N and the Cd concentration had no significant impact upon shoot biomass for either species. The N form and Cd level in solutions only influenced the root biomass of *S. nigrum*, with biomass at 15 µM Cd 18% lower when supplied with NO_3_^−^ than when supplied with NH_4_^+^. Moreover, increasing the Cd concentration in the solution from 5 to 15 µM decreased root biomass by 12% when supplied with NH_4_^+^ and by 29% when applied with NO_3_^−^ (*P*<0.05). Furthermore, the root length and root surface area per plant were 16–40% greater when supplied with NH_4_^+^ than with NO_3_^−^, irrespective of the Cd concentration in the nutrient solution (except for the root surface area of *C. rossii* at 15 μM Cd) (Supplementary Table S2).

### Plant uptake and tissue Cd concentrations

**Fig. 2.**
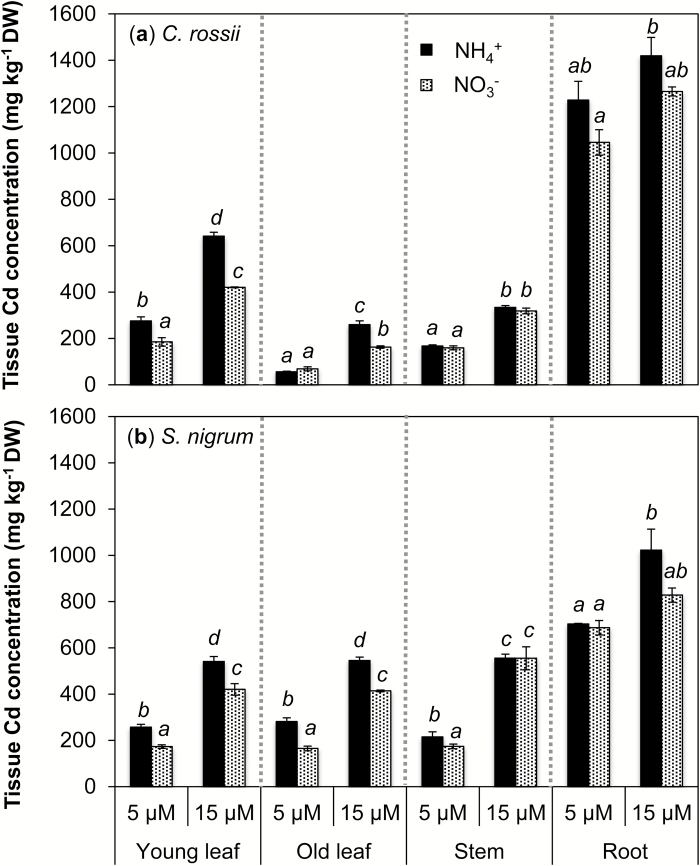
Effects of N form supplied on Cd concentrations in the different tissues of *Carpobrotus rossii* (a) and *Solanum nigrum* (b) grown for 14 d in solutions containing Cd at a concentration of either 5 or 15 µM. Bars represent the standard errors (*n*=3). For each panel, different letters above the bars indicate significant differences among treatments for each tissue of individual species (Tukey’s test, *P*<0.05).

Plant tissues differed significantly in Cd concentration (Fig. 2). Roots had the highest Cd concentrations in both species. In the shoots, *C. rossii* had highest Cd concentrations in young leaves, followed by stems and old leaves, resulting in a two-fold difference in Cd concentration between young and old leaves. In comparison, *S. nigrum* had similar Cd concentrations in the various above-ground tissues.

For both species, shoot Cd concentrations, especially leaf Cd concentrations were significantly higher when plants were supplied with NH_4_^+^ than when supplied with NO_3_^−^ (Fig. 2). For example, Cd concentrations in leaves of *C. rossii* were 50–60% higher when supplied with NH_4_^+^ than with NO_3_^−^, irrespective of the Cd concentration in the nutrient solution (except for the old leaves at 5 μM Cd). Similarly, leaf Cd concentrations of *S. nigrum* were 50–70% (5 μM Cd) and 30% (15 μM Cd) higher in the plants supplied with NH_4_^+^ than with NO_3_^−^. In addition, stem Cd concentrations of *S. nigrum* were 23% higher in NH_4_^+^-fed than NO_3_^−^-fed plants at 5 μM Cd, but did not differ between the N forms at 15 μM Cd. In addition, NH_4_^+^ treatment tended to have higher Cd concentrations in roots of both species, although the N effect was not statistically significant for either of them.

**Fig. 3.**
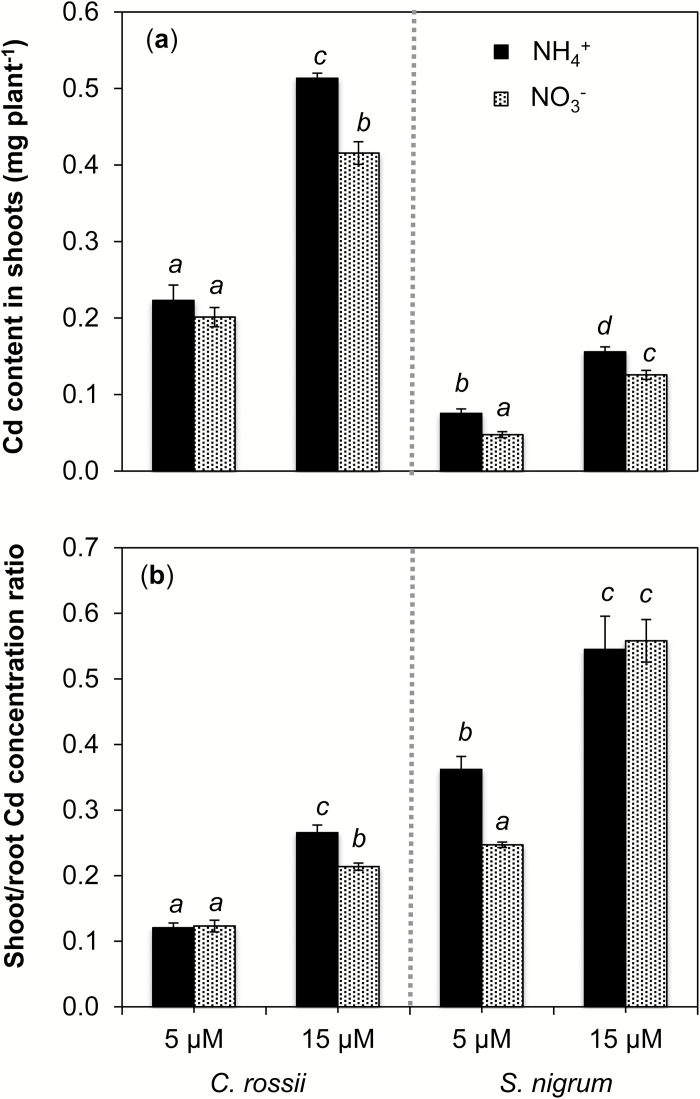
Effects of N form supplied on Cd shoot content (a) and shoot-to-root Cd concentration ratio (translocation factor) (b) of *Carpobrotus rossii* and *Solanum nigrum* grown for 14 d in solutions containing Cd at a concentration of either 5 or 15 µM. Bars represent the standard errors (*n*=3). For each panel, different letters above the bars indicate significant differences among treatments for individual species (Tukey’s test, *P*<0.05).

Accordingly, shoot Cd amounts were higher in plants supplied with NH_4_^+^ than with NO_3_^−^, although the effect of N form was greater for *S. nigrum* than *C. rossii* (Fig. 3a). Indeed, shoot Cd amounts of *C. rossii* were 20% higher in plants supplied with NH_4_^+^ than with NO_3_^−^ at 15 μM Cd (*P*<0.05), while those of *S. nigrum* were 60% (5 µM Cd) and 23% higher (15 µM Cd) in plants supplied with NH_4_^+^ than with NO_3_^−^. Overall, for a given treatment, *C. rossii* accumulated three times more Cd in shoots than *S. nigrum.* Similar trends were observed for total amounts of Cd taken up by plants and Cd uptake per unit root mass of both plant species (Supplementary Fig. S1).

For *C. rossii*, the translocation factor (i.e. the shoot-to-root concentration ratio) was not altered by the N form at 5 µM Cd, but at 15 µM Cd it was 29% higher when plants were supplied with NH_4_^+^ (Fig. 3b). In comparison, the translocation factor of *S. nigrum* was 47% higher when supplied with NH_4_^+^ than with NO_3_^−^ only at 5 µM Cd, but not at 15 µM Cd. Overall, the translocation factors were approximately twice as high for *S. nigrum* than for *C. rossii*, and were 80% higher at 15 µM Cd than at 5 µM Cd.

### Reference compounds for XAS

**Fig. 4.**
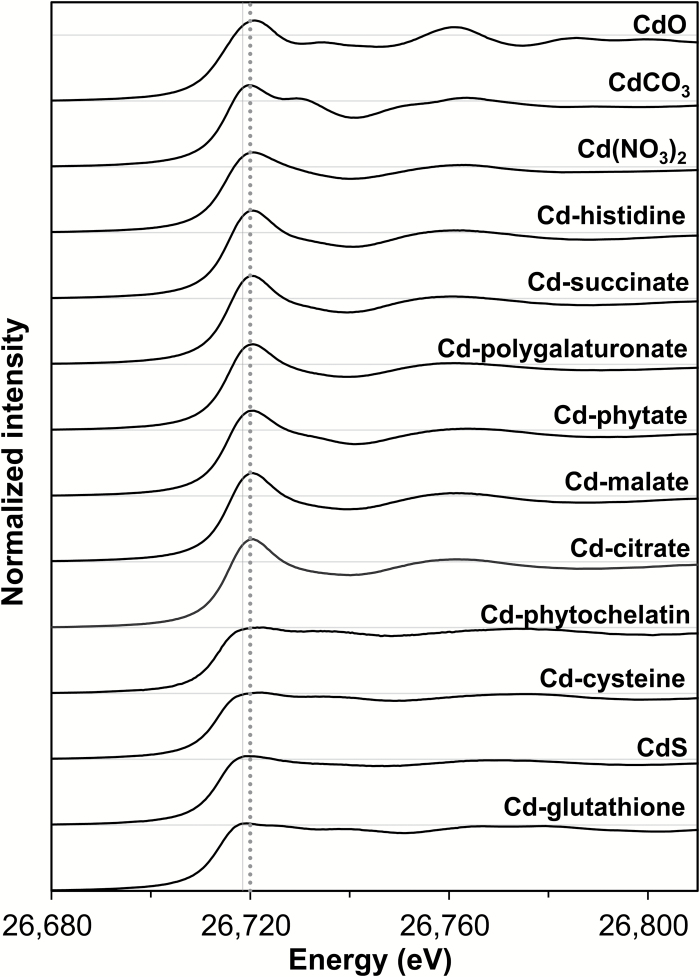
Normalized K-edge XANES spectra of the Cd standards. The horizontal grey lines represent a value of 1 for each of the normalized spectra, while the vertical grey lines represent white-line peaks of Cd-S (solid line) and Cd-OH (dotted line) standards.

The Cd K-edge XANES spectra of various Cd standard compounds were first examined visually to identify the most likely forms of Cd within the plant tissues. Given that all standards examined were prepared using Cd^2+^, the Cd K-edge XANES spectra from all standards exhibited a similar absorption edge, with white-line peaks at ca. 26 719 to 26 722eV (Fig. 4). However, the ligand to which the Cd was bound resulted in slight shifts in these white-line peaks, with Cd bound to S-containing ligands generally having white-line peaks at ca. 26 719eV, whilst the white-line peaks for Cd bound to O- or N-containing ligands were at ca. 26 720eV. Not only were slight shifts in the white-line peak observed, but differences in the shape of the spectra were also evident. For S-containing ligands, the spectra were comparatively flat and with distinctive features (Fukuda *et al.*, 2008). Furthermore, the spectra of CdO and CdCO_3_ exhibit characteristic spectral features, especially at ca. 26 729 and 26 736eV.

Although the various types of ligands influenced the spectra, those obtained for Cd(NO_3_)_2_ and for Cd complexed by various carboxyl groups (e.g. Cd-citrate, Cd-malate, Cd-succinate, Cd-polygalaturonate) were all very similar in their appearance. Likewise, the spectra for many of the S-containing ligands (such as CdS, Cd-cysteine, Cd-GSH, and Cd-PC2) were similar. Given that some spectra were largely indistinguishable from each other, hereafter, the Cd present as either free Cd [i.e. the Cd(NO_3_)_2_ standard] or as Cd complexed by carboxyl-containing compounds (i.e. citrate, malate, pectin) were grouped together and referred to as ‘Cd-OH’. Similarly, Cd-cysteine, Cd-PC2, Cd-GSH, and CdS were grouped together and referred to as ‘Cd-S’.

### Cd speciation in plant samples using XAS

**Fig. 5.**
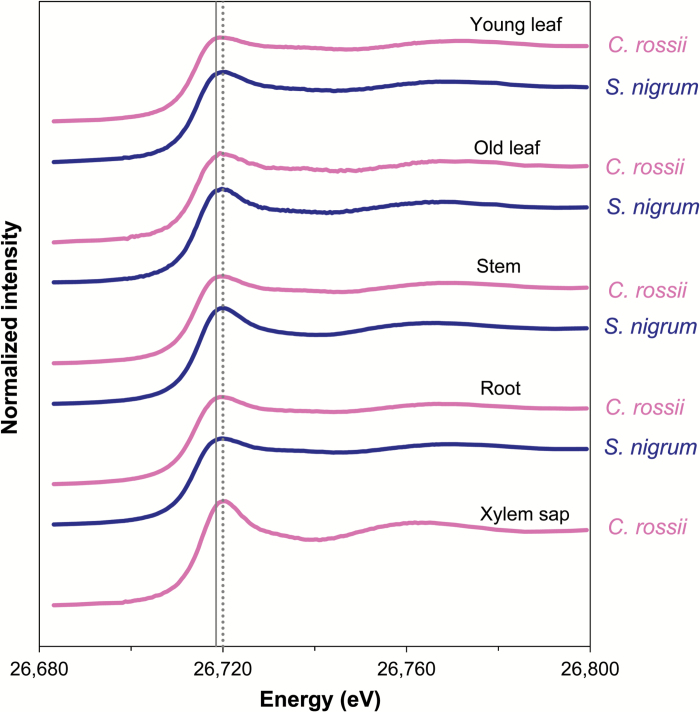
Normalized Cd K-edge XANES spectra for various tissues of two Cd hyperaccumulator plants (*Carpobrotus rossii* and *Solanum nigrum*) grown in nutrient solutions containing (NH_4_)_2_SO_4_ and 15 µM Cd. The vertical grey lines represent white-line peaks of Cd-S (solid line) and Cd-OH (dotted line) standards.

In all hydrated samples of plants grown at 15 μM Cd, the tissue Cd concentrations were sufficiently high to allow collection of XANES data with a good signal-to-noise ratio (Fig. 5). The spectra of plant samples from the 5 μM Cd treatments were collected using the freeze-dried samples due to the low Cd concentrations present. Thus the spectra of freeze-dried and frozen hydrated roots of *C. rossii* grown in 5 μM Cd were compared and were found to be similar, indicating that Cd speciation was not altered by the freeze-drying treatment (Supplementary Fig. S2). In addition, the spectra for comparable tissues of plants exposed to different Cd levels showed strong similarities, and the LCF revealed that the Cd levels in the solution did not alter Cd speciation in plants.

With regards to *C. rossii*, the spectra for both root and shoot tissues were visually similar to that of Cd-S, whilst the spectra of xylem sap clearly differed (Fig. 5). Indeed, LCF predicted that the majority (60–91%) of Cd in all tissues (other than the xylem sap) was associated with S-containing ligands, while Cd-OH was the dominant form (87–95%) in xylem sap (with the remaining 5–13% of Cd in the xylem sap associated with S) (Table 1). Although Cd-S dominated in all tissues of *C. rossii* other than the xylem sap, there was generally a higher proportion (ca. 90%) of S-bound Cd in young leaves than in old leaves and stems (ca. 60–80%). Furthermore, the spectra were visually similar for comparable tissues irrespective of the N form, indicating that the N-form did not alter Cd speciation, although LCF subsequently predicted that the proportion of Cd-OH in root tissues, xylem sap, and stems of NO_3_^−^-fed plants was ca. 10% higher than that in NH_4_^+^-fed plants (Table 1).

**Table 1.** The predicted speciation of Cd in various tissues of *Carpobrotus rossii* and *Solanum nigrum* grown for 14 d in nutrient solutions containing 15 μM Cd, as calculated using linear combination fitting (LCF) of the K-edge XANES spectra

Treatment	*C. rossii*			*S. nigrum*		
	Cd-S (%)	Cd-OH (%)	*R*-factor	Cd-S (%)	Cd-OH (%)	*R*-factor
**Young leaf**						
(NH_4_)_2_SO_4_	89 (0.8)	11 (1.2)	0.00012	68 (0.6)	32 (0.9)	0.00006
KNO_3_	91 (0.8)	9 (1.7)	0.00011	69 (1.0)	31 (0.6)	0.00006
**Old leaf**						
(NH_4_)_2_SO_4_	75 (0.8)	25 (1.4)	0.00010	55 (1.0)	45 (1.0)	0.00019
KNO_3_	75 (1.1)	25 (1.3)	0.00020	44 (1.0)	56 (1.0)	0.00018
**Stem**						
(NH_4_)_2_SO_4_	79 (0.8)	21 (2.2)	0.00013	45 (0.8)	55 (0.8)	0.00012
KNO_3_	60 (0.8)	40 (1.6)	0.00010	46 (0.8)	54 (0.8)	0.00010
**Root**						
(NH_4_)_2_SO_4_	80 (1.0)	20 (1.5)	0.00017	83 (0.6)	17 (1.1)	0.00006
KNO_3_	68 (0.6)	32 (1.6)	0.00008	85 (1.0)	15 (1.0)	0.00005
**Xylem sap**						
(NH_4_)_2_SO_4_	13 (0.9)	87 (1.9)	0.00014			
KNO_3_	5 (0.7)	95 (1.8)	0.00010			

The values in brackets show the percentage variation in the calculated values. The goodness of fit is indicated by the *R*-factor: *R*-factor = Σ_*i*_ (experiment – fit)^2^ ⁄ Σ_*i*_ (experimental)^2^, where the sums are over the data points in the fitting region.

Overall, the proportion of Cd-OH was higher in the shoots of *S. nigrum* (31–56%) than that observed for the shoots of *C. rossii* (9–40%). As observed for *C. rossii*, the proportion of Cd associated with S-containing ligands in *S. nigrum* differed between tissues, with higher levels in young leaves (ca. 70%) than in old leaves and stems (44–55%). Finally, it was again observed for *S. nigrum* that the form of N supplied did not influence Cd speciation in any tissues.

## Discussion

### Ammonium enhances Cd uptake and translocation

The present study did not support our hypothesis that the supply of NO_3_^−^, compared to NH_4_^+^, would increase Cd uptake in these two plant species. Instead, NH_4_^+^ increased Cd uptake and translocation to the shoots. It is evident that supply with NH_4_^+^ increased both the shoot Cd concentration, especially that in the leaves, and Cd accumulation in shoots, relative to that observed when supplied with NO_3_^−^. This increased Cd concentration when supplied with NH_4_^+^ was not due to a ‘dilution’ effect, because plant growth and biomass production were similar for the two N forms.

Furthermore, the difference in shoot Cd uptake between the two N forms did not result from changes in solution pH or solution Cd speciation, because buffered solution pH was constant and Cd speciation was similar for both the NH_4_^+^ and NO_3_^−^ treatments (Supplementary Table S3). With regards to the different compositions of the two N-treated solutions, the potential (*Ψ*_0_^o^) and the Cd^2+^ activity ({Cd^2+^}_0_^o^) at the cell membrane surface were calculated according to Wang *et al.* (2011), and only differed slightly between the NH_4_^+^ and NO_3_^−^ treatments (Supplementary Table S4). Thus, it appears that the increased uptake and accumulation of Cd in solutions containing NH_4_^+^ were not the result of altered bioavailability of Cd or plant growth, but instead due to other processes that influenced Cd accumulation, including Cd uptake, partitioning within the root, and efficiency of translocation from root to shoot (Clemens *et al.*, 2002; Clemens, 2006).

The higher Cd accumulation in the shoots of NH_4_^+^-fed plants could be partially attributed to the higher Cd uptake by the roots. It was evident that supply with NH_4_^+^ increased not only the total Cd uptake but also the Cd uptake per unit of root biomass in both plant species (Supplementary Fig. S1). There are a number of possible reasons for this. Firstly, it is known that N form can alter root morphology. In the present study, although the N form did not affect root biomass of *C. rossii*, root length and surface area were greater when supplied with NH_4_^+^ than when supplied with NO_3_^−^ (Supplementary Table S2), which is consistent with the findings of Liu *et al.* (2015). It has been reported previously that root development is altered depending upon the N form supplied, with NH_4_^+^ accelerating cell division and subsequent root branching (Bloom *et al.*, 2002). Thus, the NH_4_^+^-fed plants in the present study might have had a higher capacity to acquire Cd. Secondly, the regulation of Zn transporters involved in Cd uptake might potentially be enhanced in roots of NH_4_^+^-fed plants. The uptake of Cd is likely to occur through ZIP transporters (Zn/Fe-regulated transporter-like proteins) and it has been shown that increasing concentrations of tissue N can enhance the abundance of Zn-uptake transporters in the root (Erenoglu *et al.*, 2011). Considering the higher N concentration in the roots of NH_4_^+^-fed plants (Rosen *et al.*, 1990; Hassan *et al.*, 2005), the higher Cd and Zn uptake might be due to increased Zn transporters.

Other than this higher uptake by the roots, the higher Cd accumulation in the shoots of NH_4_^+^-fed plants could be partially attributed to an increased efficiency of Cd translocation to the shoots. This was evidenced by examination of translocation factors, with these being higher in NH_4_^+^- than NO_3_^−^-fed plants. This is also consistent with the observation that the Cd concentration of the xylem sap in NH_4_^+^-fed plants was 20% higher than in NO_3_^−^-fed plants (11.51 and 9.03mg L^−1^ at 15 μM Cd, respectively). Xylem loading of Cd has been shown to be mediated by the P-type ATPase transporter *AtHMA4* in different plant species such as *Arabidopsis thaliana*, *A. halleri*, *N. caerulescens* and *S. nigrum*, and overexpression of *AtHMA4* enhances Zn and Cd levels in leaves (Verret *et al.*, 2004; Verbruggen *et al.*, 2009; Xu *et al.*, 2012). The addition of NH_4_^+^, compared to NO_3_^−^, might also elevate the expression of the Cd transporter *AtHMA4* and then increase Cd translocation, but this still needs further study.

Our finding that NH_4_^+^ increased Cd uptake and accumulation in both *C. rossii* and *S. nigrum* is consistent with that observed in lettuce (*Lactuca sativa*) (Florijn *et al.*, 1992), but differs from that reported for tomato (*Solanum tuberosum*) and the hyperaccumulators *S. plumbizincicola* and *N. caerulescens*, where NO_3_^−^ increased Cd accumulation in shoots (Xie *et al.*, 2009; Luo *et al.*, 2012; Hu *et al.*, 2013). There are several possible ways to explain the apparent discrepancies between these studies. First, plant species differ in their growth response to N form. In the present study, plant biomass was not affected by N form, consistent with the result of Liu *et al.* (2015) when *C. rossii* was grown in an acid soil. In other studies, however, NH_4_^+^ significantly decreased the growth of *Solanum lycopersicum*, *S. plumbizincicola* and *N. caerulescens* (Xie *et al.*, 2009; Luo *et al.*, 2012; Hu *et al.*, 2013), with this decreased growth potentially altering Cd uptake. Second, plant species differ in their mechanisms of Cd detoxification. For example, in *S. alfredii* (which belongs to same genus as *S. plumbizincicola*) and *N. caerulescens* Cd is mainly co-ordinated with O-ligands (Küpper *et al.*, 2004; Tian *et al.*, 2011), whilst in *C. rossii* and *S. nigrum* Cd was mainly associated with S-ligands. Thus, it may not be unexpected that the effects of N form differ between these species, given that NO_3_^−^ nutrition enhances the concentration of organic acids (O-ligands) (which are important for Cd uptake and tolerance in *S. plumbizincicola* and *N. caerulescens*) whilst NH_4_^+^ nutrition increases amino acids and proteins (with Cd-S compounds important in *C. rossii* and *S. nigrum*). Third, plant species may differ in the mechanisms used to balance ion uptake when different N forms are applied. In previous studies, NO_3_^−^-fed plants accumulated more cations (including Cd) because of the antagonistic effects between NH_4_^+^ and Cd^2+^ (Xie *et al.*, 2009; Hu *et al.*, 2013), or the higher expression of *IRT1* in NO_3_^−^-fed plants than in NH_4_^+^-fed plants (Luo *et al.*, 2012). However, in the present study, significantly higher concentrations of Zn and Cd were found in NH_4_^+^-fed plants, with concentrations of other cations, including Fe, not affected by N form (Supplementary Table S5).

### Nitrogen form does not affect Cd speciation

Nitrogen form did not greatly affect Cd speciation in the plant tissues irrespective of treatment, except that the proportions of Cd-S in some tissues of *C. rossii* were slightly lower in NO_3_^−^-fed plants (Table 1). These results indicate that the increased accumulation of Cd when supplied with NH_4_^+^ could not be ascribed to alterations in Cd speciation. This observation differs from what has previously been proposed, i.e. that differences in Cd concentrations and translocation in plants supplied with different N forms might result from differences in metabolites, such as organic acids and amino acids. For example, it is known that NO_3_^−^ nutrition increases the concentration of organic acids in plants in order to maintain the intercellular pH (Marschner, 2011). Our observation that the N form did not alter speciation is similar to previous observations for the Zn-hyperaccumulator *N. caerulescens* that N form did not alter Zn speciation in different tissues, despite NO_3_^−^ enhancing the production of organic acids (Monsant *et al.*, 2011; White-Monsant and Tang, 2013). Therefore, the formation of Cd–organic acid complexes in plants may not depend on the concentrations of organic acids, but rather on efficient tonoplast transporters to facilitate Cd transport from the cytoplasm into vacuoles (Ueno *et al.*, 2005).

### Plant species and tissues differ in their Cd speciation

Although Cd-S was the dominant Cd species in the shoots of both species, the proportion of Cd-S in all the above-ground tissues was generally higher for *C. rossii* (60–90%) than for *S. nigrum* (45–70%) (Table 1). Thus, O-containing ligands tended to be more important in *S. nigrum* than in *C. rossii* – this observation for *S. nigrum* being similar to that reported previously (Sun *et al.*, 2006). These differences are not unexpected, with Cd speciation reported to differ widely between species (Castillo-Michel *et al.*, 2009; Tian *et al.*, 2011; Huguet *et al.*, 2012; Lefèvre *et al.*, 2014) and even between ecotypes of the same species (Ebbs *et al.*, 2009).

Not only did Cd speciation differ between species, but it also differed between tissues. Firstly, Cd was clearly bound to S-ligands in the roots of both species, with similar observation reported previously for other species, including corn (*Zea mays*), Indian mustard (*Brassica juncea*), and *A. thaliana* (Salt *et al.*, 1995; Isaure *et al.*, 2006; Castillo-Michel *et al.*, 2009). The high proportion of Cd-S in roots might explain the greater Cd concentration in roots than in shoots in both species, as Cd translocation was restricted by its complexation with strong ligands and its sequestration in the vacuole of root cells. Secondly, the low proportion of Cd-S in the stems of both species is inconsistent with the finding of Küpper *et al.* (2004) who found a high concentration of Cd-S in stems of *N. caerulescens* and proposed that Cd was transported as PC or MT complexes. However, in the present study, analyses of the xylem sap of *C. rossii* showed that the free Cd^2+^ ion dominated in the xylem sap, although concentrations in other aerial tissues were low. This indicates that Cd was transported as either the free ion or complexed with carboxyl groups before being chelated by S-containing compounds, which may explain the low Cd-S proportion in stems. Moreover, the importance of free hydrated Cd^2+^ ions and/or Cd-O/N complexes for Cd translocation through xylem sap has also been found previously in other Cd-hyperaccumulating and non-hyperaccumulating species (Salt *et al.*, 1995; Ueno *et al.*, 2008; Hazama *et al.*, 2015). Thirdly, young leaves tended to have a higher proportion of Cd bound to S-containing ligands than did old leaves. This decrease in Cd-S with increasing leaf age has also been reported in *N. caerulescens* (Küpper *et al.*, 2004). The higher proportion of Cd-S in young than old leaves coincided with the accumulation of more Cd in the epidermal cells of young leaves but in the mesophyll cells of old leaves, both of which would protect the highly sensitive metabolic processes in the young leaves from Cd^2+^ damage (Küpper *et al.*, 1999; Koren *et al.*, 2013).

## Conclusions

This study used synchrotron-based XAS to investigate how the N form in the nutrient supply influences Cd speciation and subsequent Cd accumulation in two newly defined Cd hyperaccumulators, which had not been examined previously. It has been demonstrated that the supply of NH_4_^+^, relative to NO_3_^−^, increased the shoot Cd accumulation by 30% in two native Australian plant species, *C. rossii* and *S. nigrum*, when grown in nutrient solution. Interestingly, this increase in Cd accumulation did not result from changes in Cd speciation within the tissues. Regardless of this, Cd speciation differed between the species and between tissues, with a higher proportion of Cd-S in the shoots of *C. rossii* than for *S. nigrum*, and more Cd being stored as Cd-S in roots and shoots but translocating as Cd-OH in the xylem sap. In addition, *C. rossii* accumulated three times more Cd in shoots than *S. nigrum*. Thus, the application of NH_4_^+^-based fertilizers would potentially favor phytoextraction of Cd by *C. rossii* and *S. nigrum*, and *C. rossii* is a better candidate for phytoextraction in Cd-contaminated soils. However, further work is required to confirm these findings, including experiments in soil- and field-based conditions.

## Supplementary data

Supplementary data are available at *JXB* online.

Figure S1. Effects of N form on the total Cd accumulation and the Cd uptake per root dry weight of plants.

Figure S2. Normalized Cd K-edge XANES spectra for freeze-dried roots and frozen hydrated roots of NH_4_^+^- fed *Carpobrotus rossii.*

Table S1. Target transformation SPOIL values of selected reference spectra obtained by principle component analysis.

Table S2. The root length and surface area per plant of the two species after 14 d of treatments.

Table S3. Speciation of Cd in the nutrient solution of different treatments.

Table S4. The calculated Cd^2+^ activities in the bulk-phase medium, the cell membrane surface potentials, and Cd^2+^ activities at the cell membrane surface in the different treatments.

Table S5. Concentrations of calcium, magnesium, zinc, and iron in shoots of plants grown for 14 d in solutions containing Cd at a concentration of either 5 or 15 µM.

Supplementary Data
