# Supplementary material for: Cadmium accumulation is enhanced by ammonium compared to nitrate in two hyperaccumulators, without affecting speciation
Source: J Exp Bot. 2016 Jul 6;67(17):5041–50. doi: 10.1093/jxb/erw270 (PMC5014155; doi:10.1093/jxb/erw270)
Supplement: Supplementary Data [file supp_67_17_5041__index.html]

Cadmium accumulation is enhanced by ammonium compared to nitrate in two hyperaccumulators, without affecting speciation — Cadmium accumulation is enhanced by ammonium compared to nitrate in two hyperaccumulators, without affecting speciation — Supplementary Data 

# Cadmium accumulation is enhanced by ammonium compared to nitrate in two hyperaccumulators, without affecting speciation

## Supplementary Data

Data files

- supplementary\_figures\_S1\_S2\_tables\_S1\_S5.pdf - Supplementary Data
