# Supplementary material for: Cadmium accumulation is enhanced by ammonium compared to nitrate in two hyperaccumulators, without affecting speciation
Source: J Exp Bot. 2016 Jul 6;67(17):5041–50. doi: 10.1093/jxb/erw270 (PMC5014155; doi:10.1093/jxb/erw270)
Supplement: Supplementary Data [file supp_erw270_supplementary_figures_S1_S2_tables_S1_S5.pdf]

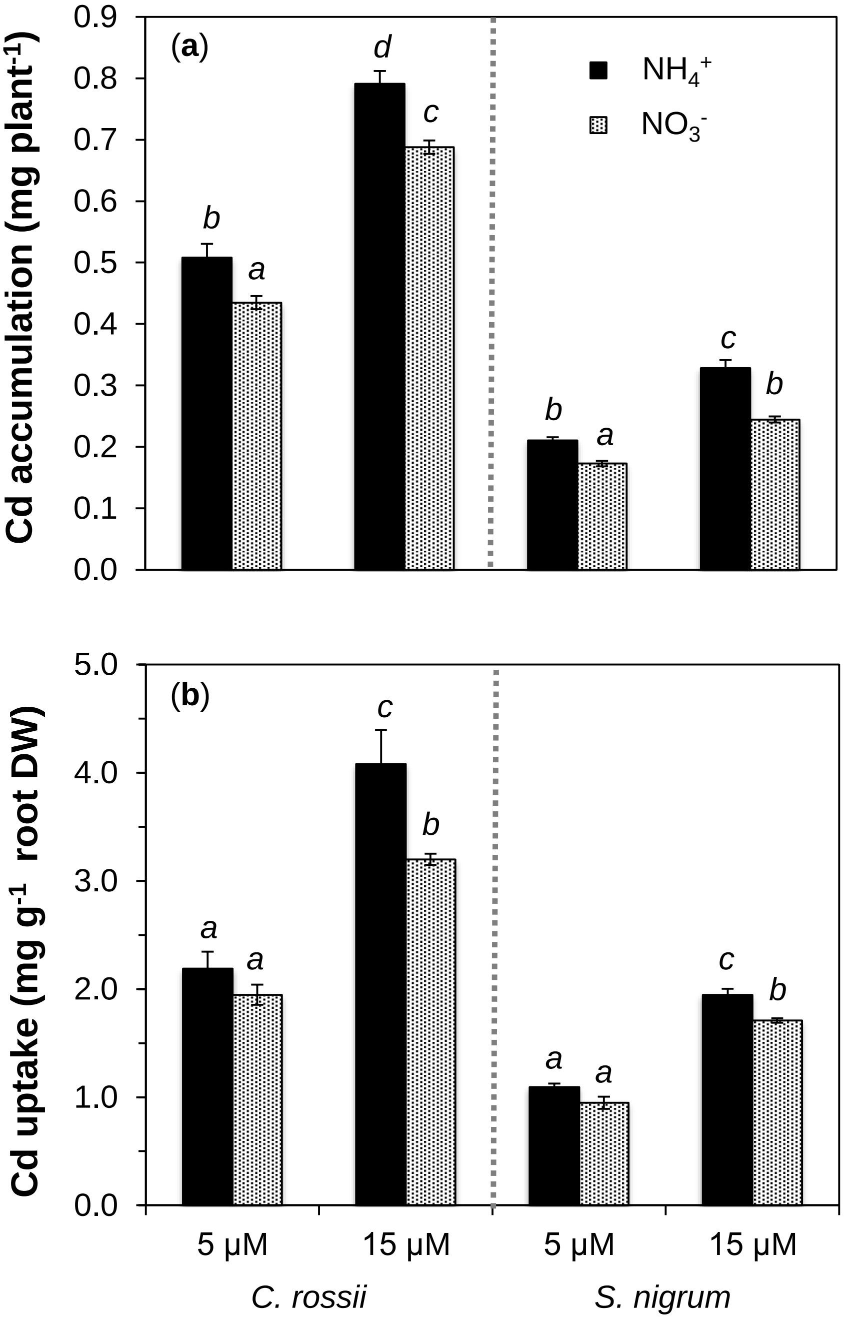

Figure S1. Effects of N form on Cd total content (a) and Cd uptake per root dry weight (b) of *Carpobrotus rossii* and *Solanum nigrum* grown for 14 d in solutions containing Cd at a concentration of either 5 or 15 µM. Bars represent the standard errors (n = 3). For each panel, different letters above the bars indicate significant differences among treatments of each tissue of individual species (Tukey's test, $P<0.05$).

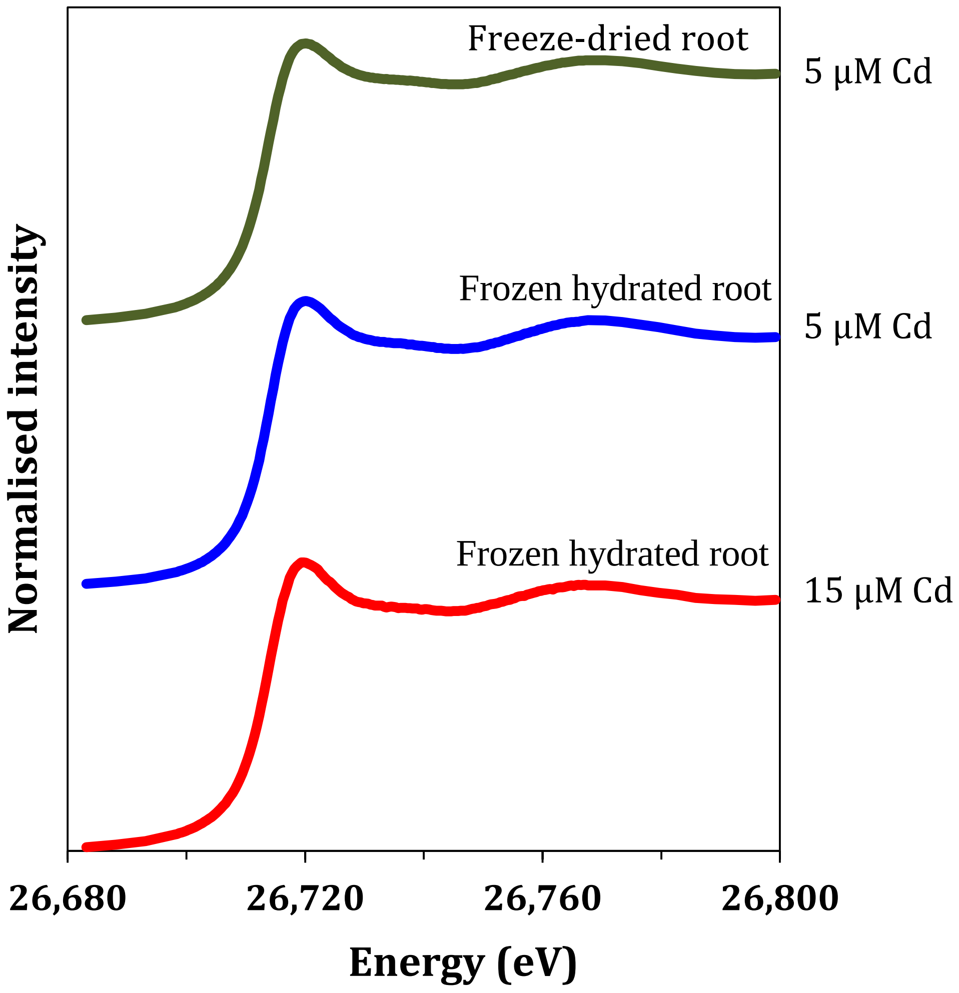

Figure S2. Normalized Cd K-edge XANES spectra for freeze-dried roots and frozen hydrated roots of NH_4^+-fed *Carpobrotus rossii*.

Table S1. Target transformation SPOIL values of selected reference spectra obtained by principle component analysis (PCA). Reference spectra are classified as excellent (SPOIL < 1.5), good (1.5-3.0), acceptable (3.0-4.5), poor (4.5-6.0), or unacceptable (> 6.0). The standard compounds with SPOIL values < 3 were included in the subsequent linear combination fitting (LCF) analyses.

	References	SPOIL VALUES
1	Cd-citrate	1.913
2	Cd-glutathione	1.977
3	Cd-malate	1.999
4	Cd-phytochelatin	2.085
5	Cd-succinate	2.113
6	$Cd(NO_3)_2$	2.402
7	Cd-cysteine	2.388
8	Cd-polygalaturonate	2.815
9	CdS	2.951
10	Cd-metallothinein	4.026
11	Cd-phytate	4.425
12	Cd-histidine	7.827
13	CdO	10.184
14	$CdCO_3$	15.046

Table S2. Root length and surface area per plant of *Carpobrotus rossii* and *Solanum nigrum* grown for 14 d in solutions containing either 5 or 15 µM Cd. Data are means ± standard errors (n = 3). The means followed by a same letter do not differ significantly within a column (Tukey's test, P<0.05).

Treatments	*C. rossii*		*S. nigrum*	
	Root length (m plant^{-1})	Root surface (cm^2 plant^{-1})	Root length (m plant^{-1})	Root surface (cm^2 plant^{-1})
NH_4^+ + 5 µM Cd	72.7 ± 3.5 c	633 ± 26 b	46.8 ± 2.7 c	617 ± 18 c
NO_3^- + 5 µM Cd	59.3 ± 2.6 ab	501 ± 25 a	38 ± 1.7 b	525 ± 14 b
NH_4^+ + 15 µM Cd	61.3 ± 1.5 b	488 ± 17 a	32.7 ± 1.5 b	525 ± 16 b
NO_3^- + 15 µM Cd	52.7 ± 1.5 a	429 ± 19 a	23.3 ± 2.0 a	417 ± 9 a

Table S3. Speciation of Cd in the nutrient solution of different treatments.

Treatments	Cd^{2+}	$CdCl^{+}$	$CdSO_4$ (aq)	$CdHPO_4$ (aq)	$CdEDTA^{2-}$
NH_4^{+} + 5 μM Cd	83.31	7.11	6.80	0.17	2.49
NO_3^{-} + 5 μM Cd	83.62	6.95	6.49	0.16	2.49
NH_4^{+} + 15 μM Cd	83.84	7.27	6.83	0.17	1.78
NO_3^{-} + 15 μM Cd	84.15	7.10	6.52	0.16	1.78

Values were estimated according to the Visual MINTEQ software and expressed as percentages of the total Cd added to the nutrient solution.

Table S4. The calculated Cd^{2+} activities in the bulk treatment solution ($\{Cd^{2+}\}_b$), the cell membrane surface potentials (Ψ_0^o), and Cd^{2+} activities at the cell membrane surface ($\{Cd^{2+}\}_0^o$) in the different treatments.

Treatments	$\{Cd^{2+}\}_b$ (μM)	Ψ_0^o (mV)	$\{Cd^{2+}\}_0^o$ (μM)
NH_4^+ + 5 μM Cd	3.0	-40.7	71
NO_3^- + 5 μM Cd	2.9	-40.3	67
NH_4^+ + 15 μM Cd	9.0	-37.9	172
NO_3^- + 15 μM Cd	8.8	-37.1	159

Table S5. Concentrations of Ca, Mg, Zn and Fe in shoots of *Carpobrotus rossii* and *Solanum nigrum* grown for 14 d in solutions containing Cd at a concentration of either 5 or 15 µM. Data are means ± standard errors (n = 3).

Plant species	Cd (µM)	N treatment	Ca (g kg^{-1})	Mg (g kg^{-1})	Zn (mg kg^{-1})	Fe (mg kg^{-1})
C. rossii	5	NH_4^+	35.2 ± 2.1	7.6 ± 0.3	150 ± 28	194 ± 22
		NO_3^-	36.1 ± 2.4	6.9 ± 0.3	153 ± 13	226 ± 36
	15	NH_4^+	46.2 ± 2.7	7.3 ± 0.2	210 ± 15	116 ± 2
		NO_3^-	39.3 ± 3.6	5.7 ± 0.3	156 ± 4	120 ± 7
S. nigrum	5	NH_4^+	18.0 ± 1.0	5.9 ± 0.2	108 ± 13	41 ± 10
		NO_3^-	20.0 ± 0.6	6.4 ± 0.1	90 ± 7	27 ± 17
	15	NH_4^+	18.9 ± 0.9	7.0 ± 0.3	94 ± 5	106 ± 1
		NO_3^-	21.9 ± 2.9	7.0 ± 0.5	79 ± 25	117 ± 24
